# Research Progress of Proton Exchange Membrane Failure and Mitigation Strategies

**DOI:** 10.3390/ma14102591

**Published:** 2021-05-16

**Authors:** Yijing Xing, Haibin Li, George Avgouropoulos

**Affiliations:** 1 State Key Laboratory of Ocean Engineering, School of Naval Architecture, Ocean & Civil Engineering, Shanghai Jiao Tong University, Shanghai 200240, China; yijingxing@sjtu.edu.cn; 2 Department of Materials Science, University of Patras, 26504 Patras, Greece

**Keywords:** proton exchange membrane, mechanical degradation, chemical degradation, degradation mitigation strategies

## Abstract

Proton exchange membrane (PEM) is critical for the efficient, reliable and safe operation of proton exchange membrane fuel cells (PEMFC). The lifetime of PEM is the main factor restricting the commercialization of PEMFC. The complexity of operating conditions, such as open-circuit/idling, dynamic load and startup-shutdown under automotive conditions, on PEMFC will cause the mechanical and chemical degradation of PEM and affect the service life of PEMFC. In order to understand the degradation behavior and durability of PEM, this paper presents an overview of the degradation failure mechanism and mitigation strategies of PEM. The mechanical and chemical degradation behavior of PEM and its causes, as well as the mitigation strategies are discussed in order to give a direction for PEM design and fuel cell system control strategy. It is proposed as a primary principle in order to further develop and promote the durability of PEM, to focus on the material improvement and system engineering.

## 1. Introduction

Proton exchange membrane fuel cells (PEMFC) is an efficient and clean energy conversion device, which can directly convert chemical energy stored in reactants into electrical energy. Membrane electrode assembly (MEA) is the core component of PEMFC, mainly including catalyst layer, gas diffusion layer and proton exchange membrane (PEM). As the key material of MEA, PEM plays multiple roles in the fuel cell operation, such as: (1) separating the gas required by cathode and anode reaction; (2) transporting protons from anode side to cathode side; (3) preventing direct electrons conduction between anode and cathode. That requires PEM not only to have excellent proton conduction and electron blocking ability, but also to have extremely low gas permeability. In the real transportation application of PEMFC stacks, the PEM needs to work in more severe working condition, which integrates water, gas, heat and electrochemical reaction. It requires that PEM should have sufficient chemical stability and mechanical strength, as well as a certain dimensional stability [1]. In fuel cells, the degeneration of catalytic layer and gas diffusion layer could lead to the decline of cell output performance. The degradation characteristics (thinning, crack and perforation) of PEM will lead to the degradation of membrane separation of the anode and cathode reaction gases. Hydrogen and air/oxygen directly mix through cross-over which could trigger hot spots and free radicals’ generation, causing irreversible damage to the fuel cells. It can be noted that the durability of membrane is a critical factor for the overall PEMFC reliability. In order to improve the durability of PEM, extensive research and development efforts are necessary. Especially for automotive applications, the required lifetime of PEMFC should be at least 5000 h and the performance degradation less than 10% in the whole service process. The degradation of PEM is considered to be the most important factor affecting the lifetime of PEMFC, and thereby requiring for PEM to have excellent durability. Therefore, proton exchange membrane reliability is highly crucial for efficient, reliable and safe operation of fuel cells. The most feasible method for evaluating the durability of PEM is to conduct long-term PEM durability evaluation tests, which is one of the reasons why the fuel cell development cycle is so long [2]. In order to better understand the origin of PEM degradation and improve its durability, this paper reviews the studies on PEM degradation behavior and mitigation strategies. Furthermore, it provides insights for the stability of PEM and MEA design, as well as strategies for system operation.

## 2. The Degradation of Proton Exchange Membrane

The degradation of PEM will affect the performance and lifetime of fuel cells [3]. In fact, the degradation of proton exchange membrane is caused by a series of complex chemical and mechanical degradation mechanisms, such as the gradual deterioration of material structure integrity, microstructure and related properties (gas permeability, proton conductivity, etc.) [4]. The fuel cell stack assembly or operation process with numerous startup-shutdown cycles create mechanical stress on the membrane, causing mechanical failure of the proton exchange membrane. Even in relatively stable environments, PEM exhibit significant chemical decay due to the permeation of reactive gases, the formation and migration of H_2_O_2_ and HO**^.^** free radical and ion pollution [5]. In the actual working environment of PEM, chemical degradation and mechanical damage always exist at the same time, which will further accelerate the degradation of membrane in a synergistic process. For example, chemical degradation causes the membrane to decompose, resulting in overall or local thinning, which grow into pinholes and cracks under the action of mechanical stress cycle. These pinholes and cracks will cause more reactive gas to penetrate, leading to more serious chemical degradation [1]. However, the existence of pinholes and microcracks does not lead to the immediate failure of the fuel cell. On the contrary, the fuel cell could still work for a long time after the pinholes are formed [6]. Table 1 reviews the degradation tests of proton exchange membrane reported in the fuel cell literature.

Membrane specific failure analysis is an important aspect of fuel cell durability research. The failure analysis usually includes the characterization of PEM structure characteristics, such as the change of the thickness and the formation of defects (e.g., cracks or perforation), as well as the changes of parameters with other membrane healthy state (fluoride ion release rate, voltage drop, H_2_ permeation, etc.). As described in reference [7], the electrochemical measurement of H_2_ permeation are used to determine the hydrogen crossover rate. Hydrogen crossover was determined by linear sweep voltammetry at room temperature. In this mode, the anode of fuel cell is filled with hydrogen as reference electrode and counter electrode, and the cathode of fuel cell is filled with nitrogen as working electrode. The hydrogen supplied to the anode permeates through the membrane and reaches the cathode, where it is electrochemically oxidized. The detection current of hydrogen molecule in fuel cell cathode oxidation is called H_2_ crossover current. The value of current is related to the amount of H_2_ permeation and could also indicate the degree of membrane attenuation.

### 2.1. The Mechanical Degration of PEM

Through the analysis of the failure forms and location of the failure membrane, the most common mechanical degradation forms of PEM include material fatigue, creep and the generation of wrinkles, delamination, pinholes or cracks. The mechanical degradation of membrane is generally considered to be the main reason for the early failure of fuel cell [8]. There are many reasons for the mechanical failure of PEM, such as improper structural design and material matching of MEA or fuel cell stack, uneven assembly or compression, changes in temperature, especially during repetitive start/stop cycles, changes in humidity, gas flow and pressure during the operation of fuel cell, which could lead to non-uniform mechanical stress or localized concentrated stress.

**Figure 1:**
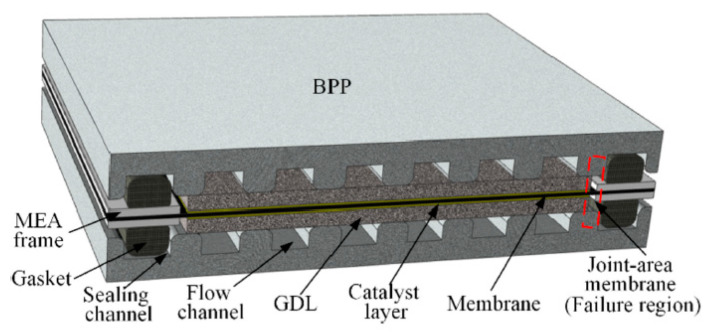
Schematic diagram of PEMFC and failure area [12].

From the failure position of MEA, the cracks or pinholes in the joint area between the MEA frame and gas diffusion layer (as shown in Figure 1) are a common mechanical failure. Wu et al. studied the degradation behavior of Nafion membrane under dry-wet and load cycling [9]. After 300 h accelerated stress test, the H_2_ crossover current suddenly increased from 7.3 mA/cm^2^ to 20 mA/cm^2^, and the open circuit voltage (OCV) decreased rapidly. The sudden increase of gas permeation along with OCV drop in cycles suggest that the pinholes or cracks appeared in some region of MEA rather than a severe loss of chemical function groups. They disassembled the failed MEA and observed significant cracks at the junction area of the PEM and the frame near the hydrogen inlet. Ye and Kang et al. simulated the stress distribution in the PEM and quantified the stress in the membrane [10,11]. Ye et al. found that there is a serious non-uniform stress area in the junction area between the PEM and the frame. When the upper and lower frames are aligned, large deformation will occur in the boundary area between the PEM and the frame, resulting in the increase of shear stress [11]. Qiu et al. presented a simulation and found that the stress-strain concentration in the junction region increased with the increase of temperature and water content, and plastic deformation even occurred in the PEM near the edge region [12]. It is also found that the stress concentration and plastic deformation in the whole active region of PEM increase rapidly when the pressure difference between anode side and cathode side exceeds 10 kPa, especially in the boundary region between membrane and frame. Therefore, the junction area between PEM and frame is the weak part of MEA, which is easy to cause membrane thinning or cracking due to mechanical stress during the short-term operation phase.

**Figure 2:**
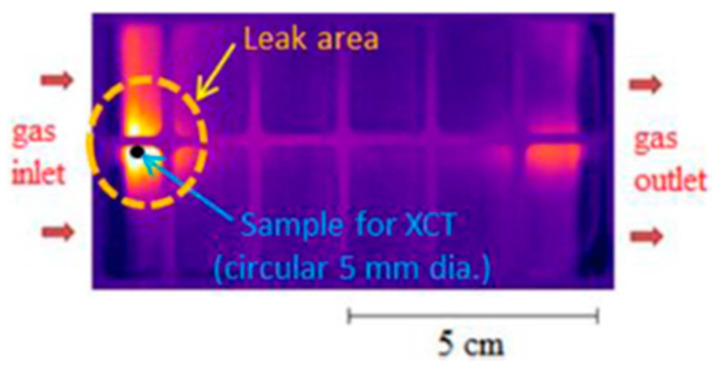
Infrared image of failure MEA in pure mechanical degradation [18].

The power demand characteristics of fuel cells determine that the PEM will experience various periodic loads, heating-cooling and start-stop cycles. In this process, the hydration state of membrane will also change dynamically, and the PEM will show anisotropic swelling and shrinking state during water absorption and dehydration. In the fuel cell’s working conditions, the in-plane expansion and contraction of the membrane are constrained by the frame and bipolar plates on both sides. This geometric constraint will produce mechanical tensile and contraction stresses on the PEM. With the long-time accumulation, the stress and strain cycle will lead to mechanical fatigue of the PEM, such as thinning, delamination, cracks and pinholes, resulting in the failure of the fuel cell [13,14]. In addition, the mechanical damage would be aggravated by inherent defects of membrane or the small assembly deviation of fuel cell stacks [15]. Alavijeh et al. [16] studied the water swelling and shrinkage behavior of PEM and the corresponding elongation through external water supplement and dehydration experiments. It was found that the mechanical creep and fatigue of the membrane was caused by accumulation of the effects of tension during the swelling and shrinkage cycles. Aindow et al. [17] simulated the stress state of PEM during humidity cycle by ex-situ mechanical fatigue experiment, drew the stress cycle failure curve to predict the mechanical durability of the membrane under humidity cycle, and predicted the residual life of PEM by comparing the fatigue test data of the initial and the degraded samples. Singh et al. [18,19] induced the pure mechanical membrane degradation by 0%–90% RH cycling accelerated test, that indicates a high crossover leakage near the gas inlet (as shown in Figure 2), which is indicator of preferentially severe mechanical degradation. At the same time, it was found that the cracks in the catalyst layer (especially the cracks on the cathode side) have a high mechanical stress concentration effect on the PEM contacting with it, which promotes the crack propagation of PEM. In addition, some theoretical simulation experiments showed that the hydration state of PEM leads to the mechanical degradation behavior of PEM [18,19], and also demonstrated that the constant and cycled stress in the cell will contribute to the development of the mechanical degradation behavior of PEM. Singh et al. [20] also proved that the crack growth behavior of membranes was related to the amplitude of stress change, temperature and relative humidity.

In the process of MEA manufacturing and fuel cell stack assembly, the material consistency, the uniformity of pressing force and the rationality of gas distribution are highly complex, which may cause the generation and variation of local stress concentration in the fuel cell. In the process of fuel cell assembly, the bipolar plate and MEA are placed in turn, and fixed together by a certain pressing force by the packaging fixture. The MEA is subjected to continuous pressure in the fuel cell. The current manual assembly process and manufacturing error of the components could be amplified under the action of continuous pressure and cumulative effect, which may accelerate the failure of membrane. Liu et al. analyzed the mechanical failure mechanism of PEM in several regions and found that the stress concentration increased with the increase of dislocation. When the manufacturing error is greater than 0.05 mm, the stress concentration will occur on the membrane at the edge of the seal ring [3]. Therefore, the influence of small deviation in the process of fuel cell parts manufacturing and assembly on the stress-strain distribution of PEM unable be ignored.

### 2.2. The Chemical Degradation of PEM

In order to improve the proton conductivity of proton exchange membrane, on the one hand, the equivalent weight value of the membrane could be reduced by increasing the number of sulfonic groups; on the other hand, the ohmic loss could be reduced by reducing the thickness of the membrane. Since the sulfonic acid group is easily attacked by free radicals, PEM with low EW value often show poor chemical durability; moreover, with the decrease of membrane thickness, the gas transport resistance decreases, which makes the reactant gas easier to penetrate, leading to chemical degradation [21,22]. Therefore, the chemical degradation of perfluorosulfonic acid PEM is not only a problem in the use of fuel cells, but also an inevitable problem in the design and development of PEM.

**Figure 3:**
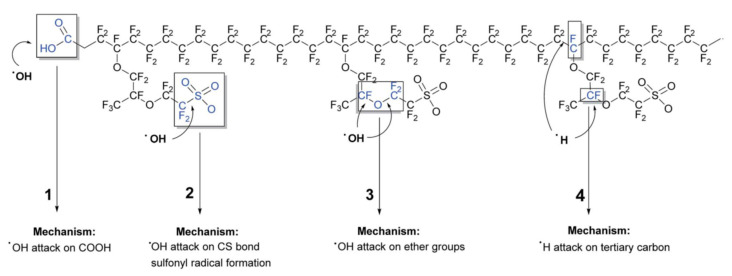
Summary of the mechanisms of radical attack on the Nafion^®^ polymer structure [25].

Compared with mechanical degradation, chemical degradation is a slow process. After a period of operation, the chemical degradation of PEM is mainly as follows: the proton conductivity decreases, and the fluorine ion release rate and gas permeability increase [23,24]. So far, the mechanism of membrane chemical degradation has been not yet fully understood. Researchers believe that chemical degradation mechanisms usually involve formation of radical chemical species by electrochemical reactions, such as hydroxy (HO^.^), hydroperoxyl (HOO**^.^**) and hydrogen (H**^.^**), which are generated as reaction by-products. The radical chemical species will attack the carboxylic acid end group sites of the primary chain and sulfonic acid groups from the side chain, leading to the chemical bonds’ cleavage and the expansion of defects [5,25]. The enlarged defect will significantly accelerate the overall rate of membrane degradation. As shown in Figure 3, there are four main mechanisms of radical attack on the Nafion polymer structure: carboxylic acid end groups; C–S bonds; tertiary carbon atoms and ether groups [25].

In situ hold at open circuit voltage (OCV hold testing) is the accepted means to accelerate membrane chemical degradation, while outside of the fuel cell environment, the Fenton reagent comprising hydrogen peroxide and ferrous ions is frequently used [25]. As described in reference [26], the chemical stability of the membrane was analyzed by Fenton test, thus stirring the membrane sample in Fenton solution at 60 °C for 2 h, and then weight the membrane sample. The oxidation resistance of the membrane is determined by the weight loss calculated by the difference between the initial weight and the final weight.

The damage of membrane caused by radical chemical species attack mainly includes: proton conductivity decrease, gas permeability increase, membrane thinning and pinhole formation [27]. It is generally believed that the chemical degradation of the membrane is related to the high temperature, high cell potential, high reaction gas pressure and low relative humidity in the fuel cell [28]. In particular, the degree of chemical degradation is more obvious at higher temperature and lower humidity [29].

**Figure 4:**
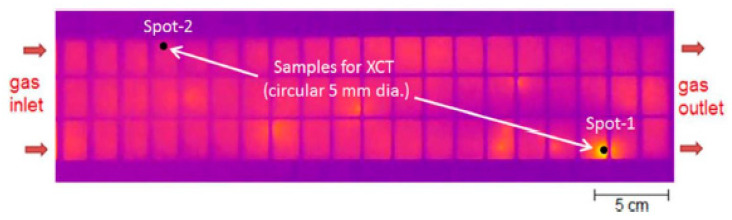
Infrared image of failure MEA in pure chemical degradation [17].

Idling condition is an important part of vehicle operation condition. In fuel cell electric vehicles, the idling conditions maintain the cell voltage close to OCV, which is known to lead to high levels of chemical degradation. Singh et al. verified the pure chemical degradation of PEM under OCV by designing experiments to ensure constant temperature, RH and flow rate of the cell. The infrared (IR) and scanning electron microscopy (SEM) images showed that the pure chemical membrane degradation proceeds generally uniform across the whole active region of MEA; however, there was a strong presence of localized extremely thin membrane regions (as shown in Figure 4) [18]. In the OCV accelerated stress test, only insignificant amounts of reactants were consumed, and the gas permeability was maximized, resulting in maximum production of radicals. Therefore, OCV accelerated stress test is often used as a special method to test the chemical degradation of PEM [30]. At the same time, the change of OCV is also a useful index to evaluate the membrane health, because the OCV decreases with the increase of gas permeability caused by membrane thinning, crack and pinhole development. Macauley et al. [31] designed an accelerated membrane durability for heavy duty fuel cells under bus related conditions. They found that elevated voltage, temperature and oxidant levels were used to accelerate membrane chemical stress, while relative humidity cycling were used to induce mechanical stress. Compared with the constant humidity condition, the relative humidity cycling could significantly reduce the membrane lifetime.

During the assembly and operation of fuel cell stack, some foreign impurities are often introduced, which will increase the chance of free radical attack on the membrane, resulting in the serious reduction of membrane life due to chemical degradation [32]. The corrosion of the fuel cell balance of plant (BOP) system materials and degradation of fuel cell stack components (bipolar plates, sealing materials, multi-metal catalysts, etc.) may release some ionic contaminants. Some ionic contaminants might transform the 4-electron path of the oxygen reduction reaction to 2-electron path, promote the generation and decomposition of H_2_O_2_, and trigger the decay of Nafion polymer molecular chain. Some ionic contaminants could exchange cations with the proton in PEM, reducing the proton conductivity of the membrane [33]. Transition metals such as Fe^2+^, Cu^2+^ and Ti^3+^ are typical pollutants produced by fuel cell BOP pipes and stack components. During the operation of the fuel cell, these transition metal ions will accumulate on PEM and catalyze H_2_O_2_ to form free radicals through Fenton reaction, thus accelerating membrane degradation [34]. Pozio et al. [35] suggested that the degradation of Nafion membrane resulted from free radicals attacking of the fluorine-containing molecular chain in the weak part of the membrane. They found that the degradation rate of Nafion membrane was different when using different metal end plates and studied the influence of metal ions (Fe^2+^, Cr^3+^, Ni^2+^) poisoning of SS316L end plate on Nafion membrane. These investigations revealed that the stainless steel is unsuitable as body material for the end plates in PEMFC. Kinumoto et al. [36] analyzed the durability of Nafion membrane in H_2_O_2_ solution containing metal ions (Li^+^, Na^+^, K^+^, Ca^2+^, Cr^3+^, Fe^2+^, CO^2+^ and Cu^2+^). They found that the presence of transition metal Fe^2+^ and Cu^2+^ ions accelerated the degradation rate of PEM, especially the degradation actions of Fe^2+^ was more significant. After 9 days, the decomposition rates of C-F bond and sulfonic group reached 68% and 33% respectively. In addition, they found that the decomposition rate of Fe^2+^ and Cu^2+^ ions on C-F bond was higher than that of sulfonic group. The decomposition and loss of sulfonic groups on Nafion membrane will reduce the proton conductivity of PEM, and the decomposition of C–F bond will lead to membrane thinning and pinhole formation, thus increasing the gas permeability. Therefore, the degradation of membrane will seriously reduce the performance of fuel cell [37]. Sun et al. [38] analyzed the mechanical properties of Nafion 212 membrane with different degrees of chemical degradation. They found that with the increase of the degree of chemical degradation, the fluorine ion release increased almost linearly, while the tensile mechanical properties and crack growth resistance of the films decreased. Moreover, the swelling behavior and water absorption of the membrane decreased due to the decrease of sulfonic acid groups. After degradation in Fenton solution for 72 h, the proton conductivity decreased by 50%.

In the working condition of fuel cell vehicle, the output power often needs to change frequently to meet the energy demand of vehicle driving. The process of power change is also known as the load changing process. This mainly brings about the changes of temperature, humidity, reactant demand and voltage, which will produce mechanical and chemical degradation on the membrane at the same time. The synergistic effects of chemical and mechanical degradation will accelerate the failure of the membrane [2]. Lim et al. [5] designed a cyclic OCV accelerated stress test to analyze the degradation process of the membrane. The OCV decreased significantly in the later stage of the experiment. After 160 h of operation, the PEM uniformly thinned, and a large number of pinholes formed. The PEM became brittle and the fluorine species in the main/side chain of PEM were lost gradually. Wu et al. [9] analyzed the degradation behavior of Nafion/ePTFE composite membrane under the combination of RH and loading cycles. In the early stage of OCV accelerated stress test, the mechanical stress caused by humidity cycle was large and uneven, which was prone to mechanical failure. At the end of OCV accelerated stress test, the PEM had serious degradation, a large number of microcracks and pinholes appeared on the membrane and the thickness of the membrane decreased from about 19 μm to 4–7 μm.

## 3. Mitigation Strategies

Under the harsh operating conditions of fuel cells, the widely used perfluorosulfonic acid proton exchange membranes tend to undergo serious mechanical and chemical degradation. Therefore, researchers try to find various ways to enhance the durability of such PEMs, in order to extend their service life in fuel cells.

### 3.1. Mitigation Strategies for Mechanical Failure of PEM

**Figure 5:**
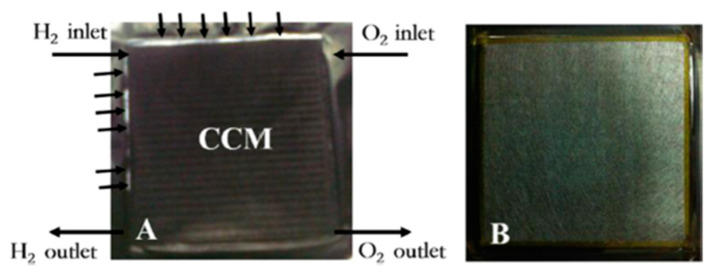
(**a**) The picture of MEA edge damage image; (**b**) The picture of MEA with edge protection [9].

Due to the strong heterogeneous stress in the junction area between frame and membrane, mechanical damage is easy to occur during operation. The structure design of MEA plays an important role in improving the mechanical durability of membrane. Ye et al. [11] observed a zone with strong nonuniform stresses in the membrane under the end edge of frame/membrane by simulation of different MEA frame materials, structures and contact behaviors. They found that the stepped frames assembly and the bonded contact behaviors have more uniform stress distributions as compared with the aligned frames assembly. The results could help the researcher to select the frame materials and design frame structures and could be applied to guide the assembly of fuel cell stacks. Wu et al. [9] found that micro cracks appeared in the PEM near the hydrogen inlet (as shown in Figure 5a) after accelerated stress test experiments for MEA without edge protection. In order to increase crack resistance, the peripheral region of MEA, especially the edge along the electrodes and membrane, should be carefully protected (as shown in Figure 5b). It has been proved that the edge protection layer could avoid sudden mechanical failure in the early stage and enable long-term operation.

As the thickness of proton exchange membrane becomes thinner, its proton conduction and water reverse diffusion ability are enhanced, but its mechanical properties such as strength and ductility are decreased, and the membrane is easy to be damaged. One way to improve the mechanical durability of PEM is to introduce more stable materials in the form of composite, so as to improve the mechanical strength and dimensional stability of PEM, and inhibit the generation of cracks and plastic deformation [39]. Lin et al. [40] immersed a sulfonated poly (amic acid) membrane in Nafion solution and then converted the sulfonated poly (amic acid) into sulfonated polyimide (SPI) by thermal imidization via solvent evaporation to prepare NF-SPI-NF multilayer membrane. Compared with native SPI and Nafion membrane, the stability and durability of NF-SPI-NF multilayer composite membrane were significantly improved [40]. The good mechanical properties of the composite membrane enable the membrane to maintain a certain strength while thinning (<20 μm) to ensure the service life of the fuel cell. Some porous reinforcement materials, such as expanded polytetrafluoroethylene (ePTFE) microporous membrane, poly (vinylidene fluoride) electrospinning microporous membrane, polyvinyl alcohol microporous membrane, have been used as the reinforcement supporting layer of composite proton exchange membrane. So far, the commercial enhanced films are Nafion XL and Gore select [41]. The microporous support layer of the reinforced membrane is located in the center of the ionomer. The microporous support layer is filled with ionomer, which helps to form a continuous proton transport channel and water transport along the thickness of the membrane. Compared with native Nafion membrane, this reinforced structure increases the yield strength and modulus of the modified PEM by two times, and reduces in-plane swelling [42]. Therefore, the mechanical reinforced membrane is relatively stable during the relative humidity cycle, and its life-time is significantly longer than that of the unreinforced membrane [43]. Tang et al. [44] compared the performance of native Nafion membrane and ePTFE/Nafion reinforce composite membrane under relative humidity cycle test conditions. The results show that the ePTFE/Nafion composite membrane are more durable than the native Nafion membrane, considering that the cyclic stress caused by swelling and shrinkage has litter effect on the reinforcement membrane. Therefore, the ePTFE microporous supporting layer could effectively inhibit the generate and propagation of cracks.

In addition to porous reinforcers, many nano-fillers are also used for mechanical reinforcement of PEM, such as carbon nanotubes [45], nanofibers [46], inorganic particles [47,48,49], clay [50] and others. The main factors affecting the preparation and processing properties of the reinforced membranes are the amount of fillers, the natural properties and the physico-chemical interaction between the fillers and Nafion matrix [51]. Wang et al. [52] prepared composite membrane by adding graphene oxide (GO) into Nafion solution. The addition of GO improved the tensile strength and dimensional stability of the membrane. The cell performance of 3 wt. % GO/Nafion composite membrane was similar to that of recast Nafion membrane, but the mechanical properties were improved. Seo et al. [53] prepared sulfonated graphite oxide (SGO) by substitution of hydroxyl group in GO into sulfonic acid group through sulfonation reaction. With the addition of SGO, the number of sulfonic acid group increases, and the proton transfer network was formed in Nafion membrane, and the Young’s modulus and tensile strength of Nafion composite membrane were improved. Vinothkannan et al. [54] used CeO_2_-TiC (titanium carbide) with high crystallinity as the filler of Nafion membrane. The thermal stability and tensile strength of composite membrane were increased to 1.4 and 1.3 times respectively due to the presence of TiC. Shaari et al. summarized the effect of additive substances such as fillers, cross-linkers, plasticizers and other additives commonly used in PEM in recent 10–15 years, focusing on the proton conductivity, mechanical properties, thermal properties, crystallinity and structure of additive modified nanocomposites. However, in the case of using additives, we should also consider the cost of additives, dispersion uniformity, environmental protection of synthesis route and the overall impact of additives on the membrane performance, so as to ensure the commercial application of the modified membrane [51].

### 3.2. Mitigation Strategies for Chemical Failure of PEM

The preparation of MEA usually employs a hot pressing process, which is conducive to the interface contact between membrane and catalytic layer [55]. During this process, the porosity, internal structure, thickness and properties of the membrane will be changed [56]. Due to the existence of micro pinholes or micropores on the membrane before hot pressing, the process of hot pressing could reduce the excess pores on the membrane and the thickness of the electrode, thus shortening the reaction path of the electrode [57]. In addition, the structure of Nafion molecules in the composite membrane may be reorganized by heat treatment during hot pressing, resulting in the decrease of gas permeability [58]. Wu et al. [9] prepared hot pressed MEA by hot pressing two gas diffusion layers and catalyst coating membrane at 110 °C and 1 MPa. After RH and loading cycle accelerated stress test experiments, it was found that the performance and durability of hot pressed MEA were better than those of unheated MEA due to the efficient proton transfer and slight chemical degradation. However, Prasanna et al. [59] found that the performance of MEA prepared by hot pressing decreased rapidly, which may be due to the high probability of water accumulation in the catalytic layer and gas diffusion layer due to the decrease of porosity of the catalytic layer after hot pressing. In order to reduce the negative effect of hot-pressing process on cell performance, the hot-pressing process parameters (hot pressing temperature, pressure and time) need to be further studied to meet the use requirements.

**Figure 6:**
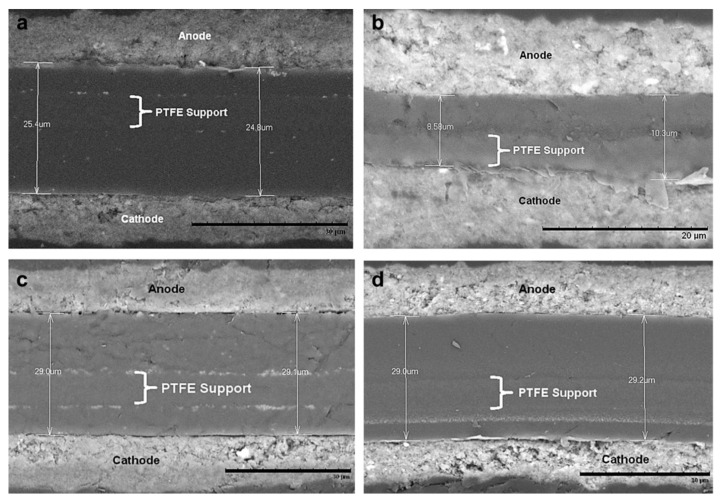
SEM images of CCM cross-sections: baseline (**a**) before and (**b**) after 500 h OCV hold test; commercial 1.0 wt. % ceria (**c**) before and (**d**) after 500 h OCV hold test [68].

**Figure 7:**
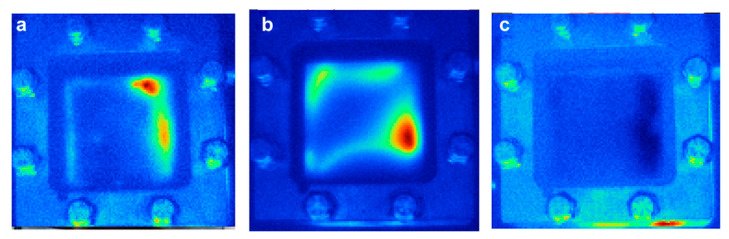
IR images of CCMs after 500 h OCV hold test: (**a**) Baseline, (**b**) synthesized 1.0 wt. % h and (**c**) commercial ceria 1.0 wt. % (red areas show higher temperature caused by reaction of hydrogen and air due to hydrogen crossover) [68].

The chemical degradation of the membrane is mainly related to the formation of peroxide free radicals during the operation of fuel cells. In order to reduce the chemical degradation of the membrane, radical scavenging materials or hydrogen peroxide decomposition could be added to the membrane to eliminate the oxidation free radicals or inhibit the generation of oxidative free radicals. By doping heteropolyacid [60], oxide, Pt catalyst [61] and other substances into PEM, the researchers found that the doped composite PEM had a certain ability to resist chemical degradation. Studies have shown that when TiO_2_, CeO_2_, MnO_2_, ZrO_2_ and other oxides are added to the membrane, the degradation rate of the membrane degradation is improved by an order of magnitude [62,63]. LaVerne et al. [64] determined the decomposition of hydrogen peroxide in aqueous suspensions of SiO_2_, Al_2_O_3_, TiO_2_, CeO_2_ and ZrO_2_ nanometer-sized particles. It was found that the decomposition of H_2_O_2_ occurs on the surface of the oxide, and the decomposition rate increases with increasing surface area of the oxide, but the number or efficiency of the reaction sites may be more important than the total surface area. It has been confirmed that the decomposition rate of H_2_O_2_ increases in the order of SiO_2_<Al_2_O_3_<TiO_2_<CeO_2_<ZrO_2_ [64]. Among these additives, cerium-based additives have been the most widely studied. Ce was an effective free radical scavenger, where the radical scavenging property was based on the reversible redox couple Ce (III) and Ce (IV) [65]. Due to the fact that the reaction rate of free radical with Ce (III) was faster than that of free radical with ionomer membrane, the chemical degradation of the membrane was alleviated [66]. Cerium could significantly improve the durability of proton exchange membrane by neutralizing free radicals before they attack the ionomer. In addition, the addition of low concentration of CeO_2_ in the membrane had little effect on the power density and ion resistance of the fuel cell. The membrane with CeO_2_ scavenger has been commercialized, such as Nafion XL [67]. Pearman et al. incorporated cerium oxide nanoparticles into perfluorosulfonic acid membranes and studied their ability to improve the in-situ membrane durability by subjecting them up to 500 h OCV hold tests. The SEM images in Figure 6a,b show that the membrane thinned considerably, from ∼25 μm to 8–10 μm, whereby the membrane on the cathode side was completely degraded, leaving the PTFE support in direct contact with the electrode. This was further supported by IR images that the MEA developed pinholes, which are visible as intense red spots in Figure 7a. Throughout the 500 h of the experiment, the commercial 1.0 wt. % MEA lost less than one percent of its total fluorine inventory and showed no change in membrane thickness (Figure 6c,d). IR images, a representative sample is presented in Figure 7c, showing no significant hydrogen crossover [68].

However, Ce^3+^ and Mn^2+^ ions migrate easily in Nafion membrane and eventually accumulate in the catalytic layer [69]. Baker et al. [67] thought that cerium migration was influenced by proton flux, potential gradient, ion concentration and water content. The most of cerium ions in commercial Nafion XL would migrate to catalytic layers of the MEA during high voltage operation at OCV, which would diminish the scavenging efficacy of cerium [67]. Therefore, the stabilization of cerium ion is importance to ensure the long-term chemical durability of the membrane. Zaton et al. and Breitwieser et al. [27,70] prepared polymer nanofiber network containing CeO_2_ particles by electrospinning technology, in which the nanofiber provides membrane mechanical reinforcement, and the attached CeO_2_ was used to eliminate free radicals to improve the membrane chemical durability. In accelerated stress test, due to the anchoring effect of polymer nanofiber, cerium without apparent migration phenomenon affected the process of degradation [27,70]. Kim et al. [71] reported a novel two-component mesoporous cerium oxide-silicon oxide composite membrane, which was proved to have higher chemical stability through Fenton experiment and H_2_O_2_ exposure experiment. Alia et al. [50] used halloysite nanotubes (HNTs) as nano-containers to encapsulate and release CeO_2_ nanoparticles. Compared with the unmodified membrane, the modified membrane containing 4 wt. % CeO_2_@HNT-NH_2_ had the same tensile properties, but improved proton conductivity and enhanced stability against radical attack.

To improve the mechanical and chemical durability of Nafion membranes, in addition to the above mentioned, adding various additives to improve the mechanical and chemical durability of Nafion membranes and optimizing the MEA preparation process, the structure modification of Nafion polymer is also a way to improve the service life of Nafion membranes. Gutru et al. collated the important literatures about the carbon nanomaterial-based PEM, the proton conductivity and methanol permeability of nanocomposite membranes with carbon nanotubes, graphene oxide and fullerene as additives were reviewed, and the effects of each filler on these properties were evaluated [72]. Schiraldi et al. thought that peroxide radicals mainly attack the hydrogen atom from residual carboxylic acid ends on PTFE backbones [73]. Such atom abstraction initiates a systematic chain oxidation reaction, which will decompose into carbon dioxide and hydrogen fluoride. Therefore, the content of the carboxylic acid ends was reduced through fluorination treatment to reduce PEM degradation and increases its durability [73]. Cross-linking is also an effective way to improve the durability of PEM. The three-dimensional cross-linking network could restrict the movement of polymer molecular chain, limit the diffusion of free radicals in PEM, maintain its structure in dry state, alleviate the stress effect caused by swelling/shrinkage of membrane during humidity cycle, and improve the mechanical and chemical stability of membrane [74,75,76]. Arslanova et al. synthesized cross-linked sulfonated polystyrene composite membrane based on commercial Nafion 115 membrane and cross-linked sulfonated polystyrene. In addition, it was found that the water content and proton conductivity of the composite membrane could be improved by cross-linking sulfonated polystyrene in the humidity range of 15%–100% RH [74].

### 3.3. Mitigation Strategies of Fuel Cell System and Operating Conditions

As mentioned above, the contaminants and impurities produced by the corrosion of BOP and fuel cell stack components would accelerate the degradation of PEM and affect the performance of fuel cell. In order to reduce the negative impact of component materials on fuel cell, the key to material selection in the future is to find BOP and fuel cell stack components with good stability and matching with the working conditions of fuel cell. Pozio et al. [35] had proved the correlation between the fluoride emission rate and the iron metal ion contamination of the end plate through experiments. Therefore, it was proposed that all the parts of the fuel cell system in contact with humidified oxygen (or air) and hydrogen should avoid releasing those elements. This stainless steel with lower Fe content (e.g., SS904L, SS310) should be much more suitable in a fuel cell system [35].

**Figure 8:**
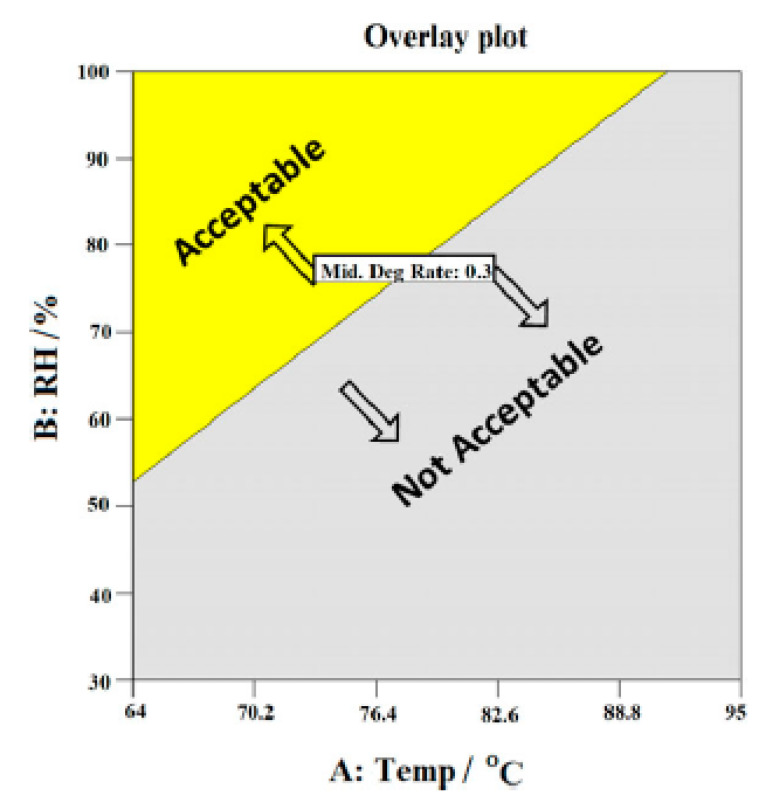
Fuel cell operation area diagram [78].

In order to further improve the durability of the membrane to achieve the life goal of automotive applications, another solution is to optimize the operating conditions of the fuel cell without upgrading the membrane materials. Under typical automotive operating conditions, dynamic load creates dynamic thermal/humidity state, changes reactant demand and induces potential cycling [2]. These factors lead to mechanical degradation of components and gas starvation. Mechanical degradation is non-uniform due to differences in the local current density and water content. Air starvation induces hydrogen pumping, thereby causing hot spots in the cathode. Lai et al. [77] found that high humidity fluctuation and/or frequent water absorption/dehydration rate could lead to high residual tensile stress in the experiment of relative humidity cycle. Reducing the intensity of relative humidity cycle could significantly delay the mechanical failure of membranes. Liu et al. [78] analysed impact of reactant gas partial pressure on membrane chemical degradation by using fluoride release rate as the assessment criterion. A strong dependency of fluoride release rate on H_2_ partial pressure was observed in the range of 20–200 kPa, and fluoride release rate increased more than 10 times when H_2_ partial pressure rised 10 times. On the contrary, there was no significant difference in fluoride release rate when O_2_ partial pressure increased from 40 to 200 kPa. Zhao et al. [78] investigated the effects of operating temperature and relative humidity on membrane durability using the OCV accelerated stress test. The results showed that the membrane degradation rate was directly proportional to the temperature and inversely proportional to the humidity. In addition, the optimum operational region was mapped without modifying membrane materials, as shown in Figure 8. The optimal region of the fuel cell could be determined by the pre-set boundary conditions. The acceptable conditions found were a temperature between 60 °C to 90 °C with 53% to 100% RH [78]. The above conclusions could provide valuable guidance for PEMFC designers and system engineers to achieve the expected service life of membrane by optimizing the operating conditions.

## 4. Conclusions and Outlook

Durability is an important factor restricting the wide commercialization of fuel cells, and the failure of proton exchange membrane is considered to be the main reason affecting the lifetime of fuel cells. As a key component of fuel cell, the service life of PEM is closely related to its initial state and working conditions. In the working conditions of fuel cell vehicle, the output power often needs to change frequently to meet the energy demand of vehicle driving. The power demand of fuel cell determines that PEM will experience frequent dynamic changes of temperature, humidity, reactant demand, current and potential, which will accelerate the mechanical and chemical degradation of PEM.

In order to improve the durability of PEM, researchers have achieved good results by adding various additives, structural modification of Nafion polymer, improvement of MEA preparation process and optimization of fuel cell system and operation conditions. Based on the existing research conclusions, the mechanical and chemical failure behaviors and durability improvement measures of PEM are summarized. It is hoped that it could provide some new ideas for the design of novel PEM and the control strategy of fuel cell system. The development of PEM durability should adhere to the principle of material improvement and system optimization in parallel: in terms of material development, PEM with better mechanical strength and oxidation resistance, stable and corrosion resistant bipolar plate and BOP components could be studied to improve mechanical and chemical durability of PEM; in terms of system optimization, the residence time of fuel cell under adverse conditions could be reduced by optimizing the system control strategy, in order to improve the service life of PEM based on the existing membrane materials. It should be noted that the design parameters of PEM (including membrane thickness, EW value, additives composition and content) are related to many characteristics of fuel cell system control strategy and need to be considered in tradeoffs.

## Figures and Tables

**Table 1:** Overview of membrane failure in lifetime test.

Researchers	AST Mode	Operating Conditions	Testing Hours	Research Findings	Ref
Healy et al.	Ex-situ degradation	Fenton reagent	24 h	The peroxyl or hydroxyl radical attack on fluorinated backbone of PEM	Healy, J. et al., 2005
Shi et al.	Ex-situ degradation	Fenton reagent	72 h	The fatigue crack propagation behavior after chemical degradation is related to the nature of the PEM	Shi, S. et al., 2020
Gubler et al.	Ex-situ degradation	Fenton reagent	//	The attack of the PFSA ionomer was assumed to proceed via weak carboxylic end-groups	Gubler, L.et al 2011
Wu et al.	RH cycling and load cycling	0% RH wet (30 min) and 100% RH dry (30 min); idle (7 min) and heavy (3 min);	300 h	Membrane thinning; Pt particles gathering along the interfaces; Fractures along the boundary of MEA;	Wu, B. et al., 2014
Panha et al.	RH cycling	0% RH wet phase (10 min) and 100% RH dry phase (40 min)	120 h	The hydrogen crossover current and fluoride ion release concentration ware increased	Panha, K. et al., 2012
Alavijeh et al.	RH cycling	Through conducting ex-situ hydration and dehydration tests	//	The accumulation of tensile stress can result in mechanical creep	Alavijeh, A.S. et al. 2019
Singh et al.	RH cycling	150% RH wet (2 min) and nearly 0% RH dry phase (2 min)	//	Membrane cracking location is shown to be strongly correlated with beginning-of-life MEA defects	Singh, Y. et al., 2019
Macauley et al.	OCV and RH cycling	the low load of only ~1A and 0%~100% RH cycling	643 h	OCV and RH cycling is more than an order of magnitude faster than for regular duty cycle testing	Macauley, N. et al., 2014
Yuan et al.	OCV	Constant current of 0.5A	1000 h	The cells with thinner membranes have lower OCV due to the higher fuel crossover	Yuan, X.-Z. et al. 2012,Yuan, X.-Z. et al. 2010
Gubler et al.	OCV	OCV hold test	//	It is likely that degradation via side-chain attack is prevalent during open circuit voltage hold tests.	Gubler, L. et al., 2011
Zhao et al.	OCV	OCV hold test	700 h	degradation rate is directly proportional to temperature and reversibly proportional to humidity	Banham, D. et al., 2015
Poizo et al.	Iron contamination	stainless steel SS316L and aluminum anticorrodal 100 end plate	2160 h	Iron contamination of membrane electrode assemblies led to polymer degradation	Pozio, A. et al., 2003
Kinumoto et al.	Iron contamination	Ion-exchange method to obtain the M^n+^Nafion (M-metal cation)	//	The presence of Fe^2+^ and Cu^2+^ greatly enhances the decomposition rate of Nafion	Kinumoto, T. et al., 2006

## Data Availability

The study did not report any data.

## References

[1] Kreitmeier S., Schuler G.A., Wokaun A., Büchi F.N. (2012). Investigation of membrane degradation in polymer electrolyte fuel cells using local gas permeation analysis. J. Power Sources.

[2] Ren P., Pei P., Li Y., Wu Z., Chen D., Huang S. (2020). Degradation mechanisms of proton exchange membrane fuel cell under typical automotive operating conditions. Prog. Energy Combust. Sci..

[3] Liu W., Qiu D., Peng L., Yi P., Lai X. (2020). Mechanical degradation of proton exchange membrane during assembly and running processes in proton exchange membrane fuel cells with metallic bipolar plates. Int. J. Energy Res..

[4] Tavassoli A., Lim C., Kolodziej J., Lauritzen M., Knights S., Wang G.G., Kjeang E. (2016). Effect of catalyst layer defects on local membrane degradation in polymer electrolyte fuel cells. J. Power Sources.

[5] Lim C., Ghassemzadeh L., Van Hove F., Lauritzen M., Kolodziej J., Wang G.G., Holdcroft S., Kjeang E. (2014). Membrane deg-radation during combined chemical and mechanical accelerated stress testing of polymer electrolyte fuel cells. J. Power Sources.

[6] Hu M., Cao G. (2014). Research on the long-term stability of a PEMFC stack: Analysis of pinhole evolution. Int. J. Hydrogen Energy.

[7] Gutru R., Peera S.G., Bhat S.D., Sahu A.K. (2016). Synthesis of sulfonated poly(bis(phenoxy)phosphazene) based blend membranes and its effect as electrolyte in fuel cells. Solid State Ion..

[8] El-Kharouf A., Chandan A., Hattenberger M., Pollet B.G. (2012). Proton exchange membrane fuel cell degradation and testing: Review. J. Energy Inst..

[9] Wu B., Zhao M., Shi W., Liu W., Liu J., Xing D., Yao Y., Hou Z., Ming P., Gu J. (2014). The degradation study of Nafi-on/PTFE composite membrane in PEM fuel cell under accelerated stress tests. Int. J. Hydrogen Energy.

[10] Kang H., Zhan Z.-G., Yang X.-F., Zhang Z.-B., Shi J.-R., Jiang P.-X., Pan M. (2020). Numerical study on the stress concentration phenomenon in the membranes of PEMFCs in an assembled state. J. Energy Eng..

[11] Ye D.H., Zhan Z.G., Lee Y.J., Tu Z.K., Zhang Y., Pan M. (2013). Effects of frame materials and structures on stress concentration of membrane electrode assembly of PEMFCs. Fuel Cells.

[12] Qiu D., Peng L., Liang P., Yi P., Lai X. (2018). Mechanical degradation of proton exchange membrane along the MEA frame in proton exchange membrane fuel cells. Energy.

[13] Uchiyama T., Kato M., Yoshida T. (2012). Buckling deformation of polymer electrolyte membrane and membrane electrode assembly under humidity cycles. J. Power Sources.

[14] Rikukawa M., Sanui K. (2000). Proton-conducting polymer electrolyte membranes based on hydrocarbon polymers. Prog. Polym. Sci..

[15] Panha K., Fowler M., Yuan X.-Z., Wang H. (2012). Accelerated durability testing via reactants relative humidity cycling on PEM fuel cells. Appl. Energy.

[16] Alavijeh A.S., Bhattacharya S., Thomas O., Chuy C., Yang Y., Zhang H., Kjeang E. (2019). Effect of hygral swelling and shrinkage on mechanical durability of fuel cell membranes. J. Power Sources.

[17] Aindow T., O’Neill J. (2011). Use of mechanical tests to predict durability of polymer fuel cell membranes under humidity cycling. J. Power Sources.

[18] Singh Y., Orfino F.P., Dutta M., Kjeang E. (2017). 3D failure analysis of pure mechanical and pure chemical degradation in fuel cell membranes. J. Electrochem. Soc..

[19] Singh Y., White R.T., Najm M., Haddow T., Pan V., Orfino F.P., Dutta M., Kjeang E. (2019). Tracking the evolution of mechanical degradation in fuel cell membranes using 4D in situ visualization. J. Power Sources.

[20] Singh Y., Khorasany R.M.H., Kim W.H.J., Alavijeh A.S., Kjeang E., Rajapakse R.K.N.D., Wang G.G. (2019). Ex situ characteri-zation and modelling of fatigue crack propagation in catalyst coated membrane composites for fuel cell applications. Int. J. Hydrogen Energy.

[21] Yuan X.-Z., Zhang S., Ban S., Huang C., Wang H., Singara V., Fowler M., Schulze M., Haug A., Andreas Friedrich K. (2012). Degradation of a PEM fuel cell stack with Nafion® membranes of different thicknesses. Part II: Ex situ diagnosis. J. Power Sources.

[22] Yuan X.-Z., Zhang S., Wang H., Wu J., Sun J.C., Hiesgen R., Friedrich K.A., Schulze M., Haug A. (2010). Degradation of a polymer exchange membrane fuel cell stack with Nafion® membranes of different thicknesses: Part, I. In situ diagnosis. J. Power Sources.

[23] Healy J., Hayden C., Xie T., Olson K., Waldo R., Brundage M., Gasteiger H., Abbott J. (2005). Aspects of the Chemical Degra-dation of PFSA Ionomers used in PEM Fuel Cells. Fuel Cells.

[24] Shi S., Sun X., Lin Q., Chen J., Fu Y., Hong X., Li C., Guo X., Chen G., Chen X. (2020). Fatigue crack propagation behavior of fuel cell membranes after chemical degradation. Int. J. Hydrogen Energy.

[25] Zaton M., Rozière J., Jones D. (2017). Current understanding of chemical degradation mechanisms of perfluorosulfonic acid membranes and their mitigation strategies: A review. Sustain. Energy Fuels.

[26] Rambabu G., Bhat S.D. (2015). Sulfonated fullerene in SPEEK matrix and its impact on the membrane electrolyte properties in direct methanol fuel cells. Electrochimica Acta.

[27] Zaton M., Rozière J., Jones D.J. (2017). Mitigation of PFSA membrane chemical degradation using composite cerium oxide–PFSA nanofibres. J. Mater. Chem. A.

[28] Rodgers M.P., Bonville L.J., Kunz H.R., Slattery D.K., Fenton J.M. (2012). Fuel cell perfluorinated sulfonic acid membrane degradation correlating accelerated stress testing and lifetime. Chem. Rev..

[29] Zhang C., Shi S., Lin Q., Wang L., Chen X. (2019). Interplay between temperature and biaxial loading on creep behavior of per-fluorosulfonic-acid membranes. J. Power Sources.

[30] Kundu S., Fowler M., Simon L.C., Abouatallah R. (2008). Reversible and irreversible degradation in fuel cells during open circuit voltage durability testing. J. Power Sources.

[31] Macauley N., Alavijeh A.S., Watson M., Kolodziej J., Lauritzen M., Knights S., Wang G., Kjeang E. (2014). accelerated membrane durability testing of heavy-duty fuel cells. J. Electrochem. Soc..

[32] De Bruijn F.D., Dam V.A.T., Janssen G.J.M. (2008). Review: Durability and degradation issues of PEM fuel cell components. Fuel Cells.

[33] Shabani B., Hafttananian M., Khamani S., Ramiar A., Ranjbar A. (2019). Poisoning of proton exchange membrane fuel cells by contaminants and impurities: Review of mechanisms, effects, and mitigation strategies. J. Power Sources.

[34] Gubler L., Dockheer Z.S.M., Koppenol W.H. (2011). Radical (HO●, H● and HOO●) formation and ionomer degradation in polymer electrolyte fuel cells. J. Electrochem. Soc..

[35] Pozio A., Silva R., De Francesco M., Giorgi L. (2003). Nafion degradation in PEFCs from end plate iron contamination. Electrochim. Acta.

[36] Kinumoto T., Inaba M., Nakayama Y., Ogata K., Umebayashi R., Tasaka A., Iriyama Y., Abe T., Ogumi Z. (2006). Durability of perfluorinated ionomer membrane against hydrogen peroxide. J. Power Sources.

[37] Chen C., Levitin G., Hess D.W., Fuller T.F. (2007). XPS investigation of Nafion® membrane degradation. J. Power Sources.

[38] Sun X., Shi S., Fu Y., Chen J., Lin Q., Hu J., Li C., Li J., Chen X. (2020). Embrittlement induced fracture behavior and mechanisms of perfluorosulfonic-acid membranes after chemical degradation. J. Power Sources.

[39] Ramani D., Singh Y., White R.T., Wegener M., Orfino F.P., Dutta M., Kjeang E. (2020). 4D in situ visualization of mechanical degradation evolution in reinforced fuel cell membranes. Int. J. Hydrogen Energy.

[40] Lin C.-C., Lien W.-F., Wang Y.-Z., Shiu H.-W., Lee C.-H. (2012). Preparation and performance of sulfonated polyimide/Nafion multilayer membrane for proton exchange membrane fuel cell. J. Power Sources.

[41] Shi S., Weber A.Z., Kusoglu A. (2016). Structure/property relationship of Nafion XL composite membranes. J. Membr. Sci..

[42] Tang Y., Kusoglu A., Karlsson A.M., Santare M.H., Cleghorn S., Johnson W.B. (2008). Mechanical properties of a reinforced composite polymer electrolyte membrane and its simulated performance in PEM fuel cells. J. Power Sources.

[43] Grohs J.R., Li Y., Dillard D.A., Case S.W., Ellis M.W., Lai Y.-H., Gittleman C.S. (2010). Evaluating the time and temperature dependent biaxial strength of Gore-Select® series 57 proton exchange membrane using a pressure loaded blister test. J. Power Sources.

[44] Tang H.L., Pan M., Wang F. (2008). A mechanical durability comparison of various perfluocarbon proton exchange membranes. J. Appl. Polym. Sci..

[45] Liu Y.-H., Yi B., Shao Z.-G., Xing D., Zhang H. (2006). Carbon Nanotubes Reinforced Nafion Composite Membrane for Fuel Cell Applications. Electrochem. Solid State Lett..

[46] Hommura S., Kawahara K., Shimohira T., Teraoka Y. (2008). Development of a method for clarifying the perfluorosulfonated membrane degradation mechanism in a fuel cell environment. J. Electrochem. Soc..

[47] Donnadio A., Pica M., Carbone A., Gatto I., Posati T., Mariangeloni G., Casciola M. (2015). Double filler reinforced ionomers: A new approach to the design of composite membranes for fuel cell applications. J. Mater. Chem. A.

[48] di Noto V., Gliubizzi R., Negro E., Pace G. (2006). Effect of SiO2 on relaxation phenomena and mechanism of ion con-ductivity of [Nafion_(SiO2)x] composite membranes. J. Phys. Chem. B.

[49] Thayumanasundaram S., Piga M., Lavina S., Negro E., Jeyapandian M., Ghassemzadeh L., Müller K., Di Noto V. (2010). Hybrid inorganic–organic proton conducting membranes based on Nafion, SiO2 and triethylammonium trifluoromethanesulfonate ionic liquid. Electrochim. Acta.

[50] Akrout A., Delrue A., Zatoń M., Duquet F., Spanu F., Taillades-Jacquin M., Cavaliere S., Jones D., Rozière J. (2020). Immobilisation and release of radical scavengers on nanoclays for chemical reinforcement of proton exchange membranes. Membranes.

[51] Shaari N., Kamarudin S.K. (2019). Recent advances in additive-enhanced polymer electrolyte membrane properties in fuel cell ap-plications: An overview. Int. J. Energy Res..

[52] Wang L., Kang J., Nam J.-D., Suhr J., Prasad A.K., Advani S.G. (2014). Composite membrane based on graphene oxide sheets and nafion for polymer electrolyte membrane fuel cells. ECS Electrochem. Lett..

[53] Seo D.C., Jeon I., Jeong E.S., Jho J.Y. (2020). Mechanical properties and chemical durability of nafion/sulfonated graphene oxide/cerium oxide composite membranes for fuel-cell applications. Polymers.

[54] Vinothkannan M., Ramakrishnan S., Kim A.R., Lee H.-K., Yoo D.J. (2020). Ceria stabilized by titanium carbide as a sustainable filler in the nafion matrix improves the mechanical integrity, electrochemical durability, and hydrogen impermeability of proton-exchange membrane fuel cells: Effects of the filler content. ACS Appl. Mater. Interfaces.

[55] Liu C.-Y., Sung C.-C. (2012). A review of the performance and analysis of proton exchange membrane fuel cell membrane electrode assemblies. J. Power Sources.

[56] Kiiver A., Vogel I., Vielstich W. (1994). Distinct performance evaluation of a direct methanol SPE fuel cell. A new method using a dy-namic hydrogen reference electrode. J. Power Sources.

[57] Zhang J., Yin G.-P., Wang Z.-B., Lai Q.-Z., Cai K.-D. (2007). Effects of hot pressing conditions on the performances of MEAs for direct methanol fuel cells. J. Power Sources.

[58] Broka K., Ekdunge P. (1997). Oxygen and hydrogen permeation properties and water untake of Nafion 117 membrane and recast film for PEM fuel cell. J. Appl. Electrochem..

[59] Prasanna M., Cho E., Lim T.-H., Oh I.-H. (2008). Effects of MEA fabrication method on durability of polymer electrolyte membrane fuel cells. Electrochim. Acta.

[60] Motz A.R., Kuo M.-C., Horan J.L., Yadav R., Seifert S., Pandey T.P., Galioto S., Yang Y., Dale N.V., Hamrock S.J. (2018). Heteropoly acid functionalized fluoroelastomer with outstanding chemical durability and performance for vehic-ular fuel cells. Energy Environ. Sci..

[61] Hagihara H., Uchida H., Watanabe M. (2006). Preparation of highly dispersed SiO2 and Pt particles in Nafion®112 for self-humidifying electrolyte membranes in fuel cells. Electrochim. Acta.

[62] Zhao D., Yi B., Zhang H., Yu H. (2010). MnO2/SiO2–SO3H nanocomposite as hydrogen peroxide scavenger for durability improvement in proton exchange membranes. J. Membr. Sci..

[63] Trogadas P., Parrondo J., Ramani V. (2008). Degradation Mitigation in Polymer Electrolyte Membranes Using Cerium Oxide as a Regenerative Free-Radical Scavenger. Electrochem. Solid State Lett..

[64] Hiroki A., LaVerne J.A. (2005). Decomposition of hydrogen peroxide at water-ceramic oxide interfaces. J. Phys. Chem. B.

[65] Weissbach T., Peckham T.J., Holdcroft S. (2016). CeO_2_, ZrO_2_ and YSZ as mitigating additives against degradation of proton exchange membranes by free radicals. J. Membr. Sci..

[66] D’Urso C., Oldani C., Baglio V., Merlo L., Aricò A. (2014). Towards fuel cell membranes with improved lifetime: Aquivion® perfluorosulfonic acid membranes containing immobilized radical scavengers. J. Power Sources.

[67] Baker A.M., Torraco D., Judge E.J., Spernjak D., Mukundan R., Borup R.L., Advani S.G., Prasad A.K. (2015). Cerium migration during PEM fuel cell assembly and operation. ECS Trans..

[68] Pearman B.P., Mohajeri N., Brooker R.P., Rodgers M.P., Slattery D.K., Hampton M.D., Cullen D.A., Seal S. (2013). The degradation mitigation effect of cerium oxide in polymer electrolyte membranes in extended fuel cell durability tests. J. Power Sources.

[69] Banham D., Ye S., Knights S., Stewart S.M., Wilson M., Garzon F. (2015). UV–visible spectroscopy method for screening the chemical stability of potential antioxidants for proton exchange membrane fuel cells. J. Power Sources.

[70] Breitwieser M., Klose C., Hartmann A., Büchler A., Klingele M., Vierrath S., Zengerle R., Thiele S. (2016). Cerium oxide deco-rated polymer nanofibers as effective membrane reinforcement for durable, high-performance fuel cells. Adv. Energy Mater..

[71] Kim J., Chung K., Lee H., Bae B., Cho E.-B. (2016). Mesoporous ceria-silica/poly(arylene ether sulfone) composite membranes for durability of fuel cell electrolyte membrane. Microporous Mesoporous Mater..

[72] Rambabu G., Bhat S.D., Figueiredo F.M.L. (2019). Carbon nanocomposite membrane electrolytes for direct methanol fuel cells—A concise review. Nanomaterials.

[73] Schiraldi D.A. (2006). Perfluorinated polymer electrolyte membrane durability. J. Macromol. Sci. Part C.

[74] Arslanova A.A., Sanginov E.A., Dobrovol’Skii Y.A. (2018). New composite proton-conducting membranes based on nafion and cross-linked sulfonated polystyrene. Russ. J. Electrochem..

[75] Hou H., Di Vona M.L., Knauth P. (2012). Building bridges: Crosslinking of sulfonated aromatic polymers—A review. J. Membr. Sci..

[76] Kim H.J., Talukdar K., Kabir M.D.L., Choi S.-J. (2018). Proton-conducting polymer membrane consisting of cross-linked poly(2-hydroxyethyl methacrylate) with Nafion® for fuel cell application. J. Nanosci. Nanotechnol..

[77] Lai Y.-H., Mittelsteadt C.K., Gittleman C.S., Dillard D.A. (2009). Viscoelastic stress analysis of constrained proton exchange membranes under humidity cycling. J. Fuel Cell Sci. Technol..

[78] Zhao N., Chu Y., Xie Z., Eggen K., Girard F., Shi Z. (2020). Effects of fuel cell operating conditions on proton exchange mem-brane durability at open-circuit voltage. Fuel Cells.

